# Morphometric Analysis of Furcation Areas of Multirooted Teeth in a Tunisian Population

**DOI:** 10.1155/2020/8846273

**Published:** 2020-09-15

**Authors:** Rym Mabrouk, Chiraz Baccouche, Nadia Frih

**Affiliations:** ^1^Dental Medicine Department, Hospital of Charles Nicolle, Tunis, Tunisia; ^2^Department of Dental Anatomy, Faculty of Dental Medicine, University of Monastir, Hospital of Taher Sfar Mahdia, Monastir, Tunisia

## Abstract

**Aims:**

The aim of the study was to evaluate the morphological characteristics of furcation of permanent molars in Tunisian population.

**Materials and Methods:**

One hundred and four extracted maxillary and mandibular permanent molars were included in this study; comprising 34 maxillary first molars, 18 maxillary second molars, 33 mandibular first molars, and 19 mandibular second molars. For each tooth, the vertical dimension of the root trunk, root length, and interradicular space width were assessed with a micrometer caliber. Different types of root trunk in maxillary and mandibular molars were also analyzed. Statistical analysis was performed using a *t*-test.

**Results:**

Root length decreased from the first to the second molars. This decrease seems to be pronounced at mandibular molars. The most observed root trunk type was type B, with a prevalence of 67.30% in maxillary molars and 51.92% in mandibular molars. The root trunk length increased from the first to the second molars in both maxillary and mandibular. The average width of the interradicular spaces varied on the same tooth and between the teeth.

**Conclusion:**

This study provides epidemiological data about molars root length, interradicular space, and root trunk vertical dimension in a Tunisian population that could help clinicians in periodontal and endodontic therapy.

## 1. Introduction

Furcation is defined by the glossary of periodontal terms as “the anatomic area of the multirooted tooth where the roots diverge” and “furcation invasion refers to the pathologic resorption of bone within a furcation” [1, 2].

Furcation morphology of multirooted teeth has been addressed extensively in the literature. There are some anatomical variations that contribute to the etiology and the compromised prognosis of furcation involved teeth. These factors include furcation entrance width, root trunk length, root concavities, enamel projections, and enamel pearls which influence the onset and progression of periodontal disease as well as the development of interradicular lesions [3–5].

Furcation areas present some of the greatest challenges of the success of periodontal therapy [4]. It has been demonstrated that many variables could alter the oral environment, such as the presence of osseointegrated implants [6], orthodontic appliances [7], or topographic hard-to-reach areas [8].

Many studies assessed the influence of topographic anatomy of molars on periodontal therapy and have shown that inaccessibility of these areas for cleaning as well as the narrowness of the furcation entrance makes adequate instrumentation and plaque control difficult. It leads to a lack of proper access for instrumentation and consequently, a persistence of pathogenic microbial flora [9]. A better understanding of the furcation and root surface anatomy is necessary for effective management of the furcation area [3, 4].

The purpose of this work was to assess the characteristic of furcation areas, the root trunk dimensions, and type of maxillary and mandibular molars and to analyze their influence on the diagnosis and management of molars with furcation involvement.

## 2. Materials and Methods

The sample of this study was composed of 430 multirooted teeth selected from a collection of extracted human teeth of a Tunisian population, obtained from different private clinics.

The reasons for extractions were following advanced periodontal disease, caries and endodontic infection, or orthodontic reasons.

The extracted teeth were placed in a solution of sodium hypochlorite 3% during one day, identified based on tooth morphology characteristics, and classified into four groups: first maxillary molars, second maxillary molars, first mandibular molars, and second mandibular molars [10, 11].

Only maxillary molars with three roots and mandibular molars with two roots were included in this study. Intact cementoenamel junction (CEJ) and intact crowns were also criteria of inclusion. Third molars, molars with fused or fractured roots, caries or restorations in the furcation areas were excluded.

A final sample of 104 teeth was retained and composed by 34 maxillary first molars, 18 maxillary second molars, 33 first mandibular molars, and 19 second mandibular molars. Teeth were cleaned under running water to remove debris and then disinfected in a solution of sodium hypochlorite 3%. If any calculus obscured the furcation entrances or the root trunk, this calculus was removed gently using a manual curette scaler (Periodontal scaler 651/11Ti.HL8. MEDESY s.r.l. Italy).

## 3. Morphometric Analysis

**Figure 1:**
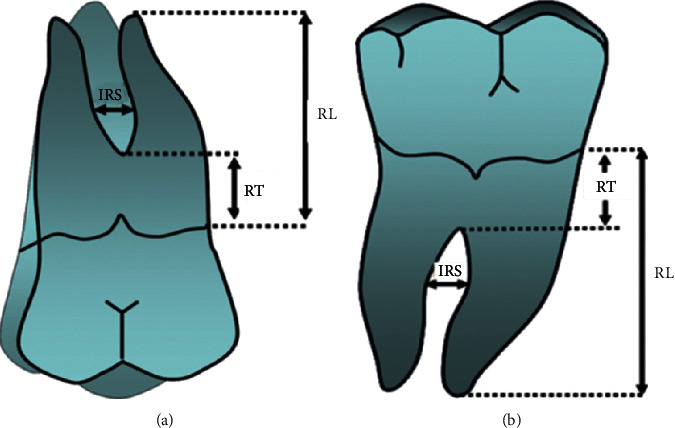
Different measured parameters in maxillary molar (a) and mandibular molar (b). RL: root length, RT: root trunk, and IRS: interradicular space.

The following parameters were measured on the selected molars:

1. The length of each root from the enamel-cementum junction to the apex of each root
2. The length of each root trunk from the enamel-cementum junction to the entrance of furcation
3. The width of the interradicular space, 1 mm apically from the furcation entrance, measured between the internal sides of the roots (Figure 1)

Measurements were carried out using a digital micrometer caliper (Fowler & NSK MAX-CAL 6” Electronic Digital LCD Caliper, Japan) with an electronic display of nearly 10^−2^. Two measurements were made separately, realized by the same operator, and the average of these measurements was calculated. If the two measures were different by more than 0.2 mm, tooth was reviewed, and the results were remeasured. All data were expressed as the mean ± standard deviation.

Using Ochsenbein's classification, the root trunk was classified into three types: A (short), B (medium), and C (long) [5, 10]. Maxillary molars with root trunks of 3 mm or less were classified as short, 4 mm trunks were classified as medium, and 5 mm or more trunks were classified as long. For mandibular molars, a short root trunk was considered to be 2 mm or less, medium root trunks were 3 mm, and long root trunks were 4 mm or longer.

For the evaluation of the variables, root length, root trunk, and interradicular space width, statistical analysis was performed using a *t*-test. Statistical analysis was performed using IBM statistical package for the social sciences statistics 21 programs (IBM SPSS statistics, Armonk, NY, USA). Correlations between root trunk length and interradicular space width, root trunk length, and root length were calculated using Pearson's correlation coefficient. The level of significance was set as *p* < 5.10^−2^_._

## 4. Results

The mean values of root length (RL), root trunk length (RTL), and interradicular space (IRS) width of the examined teeth are presented in Tables 1 and 2.  Table 3 illustrates the comparison of the different studied parameters.

### 4.1. Root Length

At maxillary molars, distobuccal root was the shortest (*p*=0.002) followed by the mesiobuccal root and the palatal root (12.32 mm, 13.17 mm, 13.38 mm), respectively (Table 1). However, this order was not always conserved as the palatal root was not always the longest one (*p*=0.747). Some molars with mesial roots longer than the palatal one were also noticed.

At mandibular molars, the mesial root was the longest (14.69 mm) followed by the distal root (13.74 mm), and this result seemed to be statistically significant (*p*=0.002) (Table 3). Root lengths decreased from the first to the second molars. This decrease seemed to be pronounced at mandibular molars.

### 4.2. Root Trunk Length

The length of the root trunk increased from the first to the second molars in both maxillary and mandibular. The order of the increasing average of root trunks was the same for the first molars and second maxillary molars: buccal, distal, and mesial (Table 2).

It can be perceived that in the Tunisian population, the mesial root trunk was the longest one on maxillary molars. The buccal root trunk was the shortest one in comparison with other root trunks in both arches (Table 3).

Table 4 shows that the most observed root trunk type was type B with a prevalence of 67.30% in maxillary molars and 51.92% in mandibular molars. The mean value of root trunk length for maxillary molars ranged from 3.96 mm to 4.90 mm, while for mandibular molars, root trunk length varied from 3.75 mm to 4.47 mm.

### 4.3. Interradicular Space Width

Table 2 lists the mean values of the width of the interradicular spaces in maxillary and mandibular first and second molars. The mean width of the interradicular spaces varied on the same tooth and between the teeth. Regarding the interradicular space dimensions of maxillary molars, the buccal interradicular space was the narrowest, followed by the mesial than the distal one (*p*=10^−3^), whereas in mandibular molars, the buccal interradicular space was larger than the lingual one.

It can also be observed that with the increasing mean of root trunk length, there was a decrease in the interradicular space width. It was interesting to note that for mandibular molars, lingual furcation was characterized by a long root trunk associated with narrower interradicular space.

## 5. Discussion

Our study was based on measurements using a micrometric caliper with an electronic display with an accuracy of 10^−2^; the reading was difficult. However, it was done by one operator which minimized the risk of error. Moreover, dental anatomy is highly variable; the random collection of teeth in our study overcame the anatomical diversity of the molars.

The finding of the present study showed that the mean length of the mesiobuccal and palatal roots, in maxillary first molars, was, respectively, 13.17 mm and 13.38 mm. The distobuccal root was the shortest. This order was the same for the second maxillary molars.

However, for mandibular first molars, the means of the mesial and distal roots were, respectively, 14.69 mm and 13.74 mm. In mandibular second molars, the root length means were 14.04 mm in mesial side and 13.34 mm on the distal side of the tooth.

These results were in accordance with those reported by Dababneh et al. [11] who found that for maxillary first molars, the mean lengths of the mesiobuccal and palatal roots were closer (12.9, 13 mm) and longer than the distobuccal root (11.9 mm), while for mandibular first molars, the means of the mesial and distal roots were, respectively, 14 and 13.5 mm. Different results were obtained by Roussa [12] for the maxillary molars who found that the distobuccal was the longest root (12.2 mm) compared to 11.3 mm and 11.2 mm for, respectively, the mesiobuccal and palatal roots. On the other hand, for mandibular molars, they found that the means for the mesial and distal roots were, respectively, 14.2 mm and 14 mm. Distal roots were found to be longer than mesial roots. These morphometric measurement variations could be attributed to geographic and ethnic differences.

The root trunk is defined as the area of the tooth extending from the cementoenamel junction to the furcation [3]. The present study showed that the most observed type was type B followed by type A and type C for both maxillary and mandibular molars. The prevalence of type C in the present study (13.46% in maxillary molars and 3.84% in mandibular molars) was higher than those reported in other studies such as those cited by Hou et al. and Dababneh et al. [11, 13].

The root trunk length increased from the first to the second molars at both maxillary and mandibular arches. This finding was in accordance with those reported by Kerns et al. [5]. The main finding of the present study was that the buccal furcation was anatomically different from the lingual, mesial, and distal furcations for all the evaluated measurements [13]. The buccal root trunk length was shorter than the mesial and distal root trunk and the mesial root trunks were the longest root trunk in maxillary molars. This data was in agreement with others founded in other studies [11, 14]; however, it disagreed with some other studies that found that the distal root trunk was longer than the mesial one [12, 15, 16].

In mandibular molars, the buccal root trunk was shorter than the lingual root trunk. This finding was in accordance with the morphometric studies of root trunk [5, 13, 17, 18] (Table 5).

Root trunk length has an important impact on the pathogenesis of the periodontal disease. This is one of the keys to anatomical factors that make molars particularly susceptible to periodontal disease [21, 22]. Short root trunk is more likely to develop early furcation involvement and attachment loss in the presence of periodontal disease because it has less surface area for periodontal attachment. Even though, once the disease is installed, reduced root trunk length tends to lead to satisfactory periodontal treatment outcomes because of its easier access [3].

On the other hand, a long root trunk makes access to the proximal furcation more difficult compared to the other sides, particularly when neighboring teeth are present. Diagnosis and treatment could be better with surgical exposure in the case of furcation involvement because of a lack of access [23, 24].

The furcation entrance measure is extremely important in anticipating the success of periodontal therapy. In this study, buccal furcation was statistically the narrowest for maxillary molars, while the lingual furcation was narrower than the buccal furcation for mandibular molars. These results were comparable to other results in other studies [13, 15, 17, 20, 25]. Narrow furcation implies an increased difficulty of access through furcation entrances for complete root debridement leading to a poor periodontal outcome. This fact seemed to be accentuated in the present study because of the long lingual root trunk in mandibular molars associated with a narrower furcation [24].

The present study showed that the mean of the interradicular space width was superior to 0.98 mm at 1 mm of the furcation entrance. This result could be a micrometric characteristic of the Tunisian population, which seemed to be similar to the dimension of standard Gracey curettes (75 to 0.95 mm). This finding indicated that the use of curettes alone might be suitable for root preparation in the furcal area.

However, the micrometric measurements of the present study were more important than those reported in several studies. In fact, Kodovic et al. [22] reported that 81% of all furcation entrances diameters were <1 mm and 58% were <0.75 mm. Sixty-three percent of maxillary molars and 50% of mandibular molars were <0.75 mm. Different findings were also reported by Castro-Rodríguez et al. where 49% of furcation entrances were found to be <0.75 mm [23].

The small number of teeth used in the present study could be considered as a limitation. In fact, the main inclusion criteria were intact crown and roots. However, most of the first and second permanent molars extracted were severely affected by decay or badly destructed which impeded tooth measurement. This fact can limit the extrapolation of the results, particularly concerning the distribution of root trunk in maxillary and mandibular molars to the entire population. Nevertheless, no survey on the anatomy of the furcation of permanent molars in the Tunisian population has been published at the time of the preparation of this study. Therefore, the results of this study may provide useful data of the micrometer measurements of permanent molars roots and the anatomy of the root trunk and furcation which can help in defining new strategies for treatment and prevention of periodontal diseases. Further studies involving a larger sample will be an essential step in improving the periodontal health status of the Tunisian citizens.

## 6. Conclusion

The data of the present study provide reference measurements for therapeutic applications in molars of the Tunisian population within the limits of the sample studied. Furcation areas of multirooted teeth are extremely complex and must be carefully understood to improve the success rate of periodontal therapy. Knowledge of root trunk characteristics and interradicular dimensions, coupled with furcation and osseous architecture, should aid the clinician in the diagnosis, management, and prognosis of periodontally involved molars.

## Figures and Tables

**Table 1:** Means ± SD (standard deviation) of root lengths of investigated teeth (mm).

Type/site of molars		First molars (mm)	Second molars (mm)
Maxillary	Mesiobuccal RL	13.17 ± 1.42	13.14 ± 1.43
	Distobuccal RL	12.32 ± 1.13	12.04 ± 1.24
	Palatal RL	13.38 ± 1.34	13.28 ± 1.41

Mandibular	Mesial RL	14.69 ± 1.05	14.04 ± 1.46
	Distal RL	13.74 ± 1.04	13.34 ± 1.30

^*∗*^RL = root length.

**Table 2:** Means ± SD (standard deviation) of root trunk length and interradicular space width of investigated teeth.

Tooth/side	Root trunk length (mm)	Interradicular space width (mm)
First maxillary molars		
Buccal	3.96 ± 0.77	1.18 ± 0.39
Mesial	4.32 ± 0.90	1.55 ± 0.45
Distal	4.00 ± 0.74	1.96 ± 0.39

Second maxillary molars		
Buccal	4.28 ± 1.06	0.98 ± 0.29
Mesial	4.90 ± 1.05	1.44 ± 0.34
Distal	4.46 ± 1.09	1.53 ± 0.2

First mandibular molars		
Buccal	3.75 ± 0.58	1.41 ± 0.32
Lingual	4.40 ± 0.67	1.29 ± 0.37

Second mandibular molars		
Buccal	3.90 ± 0.74	1.06 ± 0.25
Lingual	4.47 ± 0.94	0.96 ± 0.22

**Table 3:** Comparison of the different studied parameters of investigated teeth.

Maxillary root length		Mandibular root length
MBR	DBR = 0.002	MR DR = 0.002
	PR = 0.747	
DBR	PR = 10^−3^	
	MBR = 0.002	
PR	MBR = 0.747	
	DBR = 10^−3^	

Maxillary root trunk length		Mandibular root trunk length

MRT	BRT = 0.005	BRT LRT = 10–3
	DRT = 0.017	
DRT	MRT = 0.017	
	BRT = 0.854	
BRT	DRT = 0.854	
	MRT = 0.005	

Maxillary interradicular space width		Mandibular interradicular space width

MIRS	BIRS = 10^−3^	LIRS BIRS = 0.106
	DIRS = 0.276	
DIRS	BIRS = 10^−3^	
	MIRS = 0.276	
BIRS	MIRS = 10^−3^	
	DIRS = 10^−3^	

MBR: mesiobuccal root, DBR: distobuccal root, PR: palatal root, MR: mesial root, DR: distal root; MRT: mesial root trunk, BRT: buccal root trunk, DRT: distal root trunk, LRT: lingual root trunk; MIRS: mesial interradicular space, DIRS: distal interradicular space, BIRS: buccal interradicular space, and LIRS: lingual interradicular space.

**Table 4:** Distribution of root trunk type in maxillary and mandibular molars.

Root trunk type	Maxillary molars (%)	Mandibular molars (%)
Type A	19.23	44.23
Type B	67.30	51.92
Type C	13.46	3.84

**Table 5:** Comparison of root trunk dimension of mandibular and maxillary molars between this study and other studies.

Author/year of publication	Maxillary molars			Mandibular molars	
	MRT	DRT	BRT	LRT	BRT
The present study	4.61	4.23	4.12	4.43	3.82
Dababneh et al. [11]	4.98	4.31	3.97	4.31	3.75
Roussa [12]	3.49	4.14	3.46	3.5	2.8
Plagmann et al. [14]	4.8	4.5	4.3	4.3	3.3
Dunlap and Gher [16]	3.6	4.8	4.2		
Dunlap and Gher [16]				4.0	4.0
Rosenberg [19]	5.0	3.5	3.0		
Mandelaris et al. [20]				4.17	3.14
Kerns et al. [5]	4.7	4.7	4.1	4.3	3.3
Porciúncula et al. [21]	4.44	4.26	3.50		

MRT: mesial root trunk, BRT: buccal root trunk, DRT: distal root trunk, and LRT: lingual root trunk.

## Data Availability

The data of the assessed parameters used to support the finding of the present study are available from the corresponding author upon request.

## References

[1] American Academy of Periodontology (2001). *Glossary of Periodontal Terms*.

[2] Luigi N. (2018). *Diagnosis and Treatment of Furcation-Involved Teeth*.

[3] Al-Shammari K. F., Kazor C. E., Wang H. L. (2001). Molar root anatomy and management of furcation defects. *Journal of Clinical Periodontology*.

[4] Karthikeyan B. V., Sujatha V., Prabhuji M. L. (2015). Furcation measurements: realities and limitations. *Journal of the International Academy of Periodontology*.

[5] Kerns D. G., Greenwell H., Wittwer J. W., Drisko C., Williams J. N., Kerns L. L. (1999). Root trunk dimensions of 5 different tooth types. *The International Journal of Periodontics & Restorative Dentistry*.

[6] Al-Majid A., Alassiri S., Rathnayake N., Tervahartiala T., Gieselmann D. R., Sorsa T. (2018). Matrix metalloproteinase-8 as an inflammatory and prevention biomarker in periodontal and peri-implant diseases. *International Journal of Dentistry*.

[7] Sfondrini M. F., Debiaggi M., Zara F. (2012). Influence of lingual bracket position on microbial and periodontal parameters in vivo. *Journal of Applied Oral Science*.

[8] Pacha-Olivenza M. Á., Tejero R., Fernández-Calderón M. C., Anitua E., Troya M., González-Martín M. L. (2019). Relevance of topographic parameters on the adhesion and proliferation of human gingival fibroblasts and oral bacterial strains. *BioMed Research International*.

[9] Cobb C. M. (1996). Non-surgical pocket therapy: mechanical. *Annals of Periodontology*.

[10] De Los Rios C. M., Pustiglioni F. E., Romito G. A. (2002). Biometric study of the width, length and depth of the root trunk groove of human lower second molars. *Pesquisa Odontológica Brasileira*.

[11] Dababneh R., Samara R., Abul-Ghanam M. A., Obeidat L., Shudifat N. (2011). Root trunk: types and dimension and their influence on the diagnosis and treatment of periodontally involved first molars. *Journal of Research in Medical Sciences*.

[12] Roussa E. (1998). Anatomic characteristics of the furcation and root surfaces of molar teeth and their significance in the clinical management of marginal periodontitis. *Clinical Anatomy*.

[13] Hou G. L., Hung C. C., Tsai C. C., Chen P.-H., Yang Y.-H., Shieh T.-Y. (2003). Topographic study of extracted molars with advanced furcation involvement: furcation entrance dimension and molar type. *The Kaohsiung Journal of Medical Sciences*.

[14] Plagmann H.-C., Holtorf S., Kocher T. (2000). A study on the imaging of complex furcation forms inupper and lower molars. *Journal of Clinical Periodontology*.

[15] Cleghorn B. M., Christie W. H., Dong C. C. S. (2006). Root and root canal morphology of the human permanent maxillary first molar: a literature review. *Journal of Endodontics*.

[16] Dunlap R. M., Gher M. E. (1985). Root surface measurements of the mandibular first molar. *Journal of Periodontology*.

[17] Marcaccini A. M., Pavanelo Â., Boas Nogueira A. V., Chaves de Souza J. A., Porciúncula H. F., Cirelli J. A. (2012). Morphometric study of the root anatomy in the furcation area of mandibular molars. *Journal of Applied Oral Science*.

[18] Ochsenbein C. (1986). A primer for osseous surgery. *The International Journal of Periodontics & Restorative Dentistry*.

[19] Rosenberg M. M., Rosenberg M. M., Kay H. B., Keough B. E., Holt R. L. (1988). Furcation involvement: periodontic, endodontic and restorative interrelation ships. *Periodontal and Prosthetic Management for Advanced Cases*.

[20] Mandelaris G. A., Wang H. L., Mac Neil R. L. (1998). A morphometric analysis of the furcation region of mandibular molars. *Compendium of Continuing Education in Dentistry*.

[21] Porciúncula H. F., Zuza E. P., Da Porciúncula M. M., De Toledo B. E., Mendes A. J. (2007). Root trunk height as a risk factor for periodontal furcation involvement in maxillary first molars: an in vitro study. *Journal of the International Academy of Periodontology*.

[22] Kadovic J., Novakovic N., Jovanovic M. (2019). Anatomical characteristics of the furcation area and root surfaces of multi-rooted teeth: epidemiological study. *Vojnosanitetski Pregled*.

[23] Castro-Rodríguez Y., Sihuay-Torres K., Saenz-Velarde R., Quispe-Romero P., Valle-Armas E., Albornoz-Miranda F. (2018). Morphometric characteristics of multirooted teeth and furcation area. *Odontoestomatología*.

[24] Santana R. B., Uzel M. I., Gusman H., Gunaydin Y., Jones J. A., Leone C. W. (2004). Morphometric analysis of the furcation anatomy of mandibular molars. *Journal of Periodontology*.

[25] Pilloni A., Rojas M. (2018). Furcation involvement classification: a comprehensive review and a new system proposal. *Dentistry Journal*.

